# Non-operative treatment for simple acute appendicitis (NOTA) in children during the COVID-19 era: new lessons from the pandemic

**DOI:** 10.1038/s41598-023-46172-2

**Published:** 2023-10-31

**Authors:** Ameen Alsaggaf, Yazeed Owiwi, Mohamed Shalaby, Alaa Ghallab, Mazen Zidan, Ahmed Alawi, Nasir Bustangi, Mohammed Awad, Abdulelah Bana, Saad Al Zeair, Ahmed Afandi, Ahmed Basyouni, Ibrahim Al Nasser, Enas Raml, Enaam Raboei

**Affiliations:** 1 Pediatric Surgery Department, King Fahd Armed Forces Hospital (KFAFH), Jeddah, Saudi Arabia; 2 Pediatric Surgery Unit, Faculty of Medicine, Tanta University, Tanta, Egypt; 3 Division of Pediatric Surgery, Department of Surgery, Faculty of Medicine, King Abdulaziz University, Jeddah, Saudi Arabia; 4 Medical Reference Center, Jeddah, Saudi Arabia; 5 Radiology Department, King Fahd Armed Forces Hospital (KFAFH), Jeddah, Saudi Arabia; 6 Surgical Department, King Fahd Armed Forces Hospital (KFAFH), Jeddah, Saudi Arabia

**Keywords:** Diseases, Gastroenterology, Health care, Risk factors, Signs and symptoms

## Abstract

Coronavirus (COVID-19) was a pandemic disease that was affecting our medical and surgical daily practice badly. The surgical management of acute appendicitis was the gold standard, but new studies suggest the safety of antibiotic treatment alone. Non-operative treatment for simple acute appendicitis (NOTA) avoids surgery, the risks of general anesthesia, and long hospital stays. It also decreases the risk of exposure to coronavirus. We aimed to study the cost-effectiveness and outcome of NOTA during the COVID-19 pandemic and compared it to single-incision pediatric endo-surgery appendectomy (SIPESA). A prospective cohort study for NOTA of patients from 6 to 12 years old in the COVID-19 pandemic period from April 1st, 2020, to April 30th, 2021, patients were divided into two groups: Group S was managed by SIPESA, and Group N was managed by NOTA. Family education and assurance with detailed explanation were done for early detection of any complications, and we continue monitoring the patients until their complete recovery. Group S had 24 cases (40%), mean age 9.3 years. Group N had 36 cases (60%), mean age 9.1 years. Six cases (17%) in group N were converted to surgical management in the first 6 months of the study. The mean cost dropped from $2736/day to $400/day. The mean psychological stress for the children improved from 4.4 in April to 2 in September. The mean follow-up was 3.5 months. NOTA is a feasible, cost-effective approach, and we recommend it, as we have learned this lesson during the COVID-19 pandemic days.

## Introduction

Coronavirus (COVID-19) was a major pandemic communicable disease. The outbreak started in December 2019 and rapidly expanded within Wuhan, China. The illness spread as a global pandemic, according to the WHO, in March 2020, to the rest of the world, including the Middle East^1,2^. COVID-19 represented an uncertain challenge that could affect large numbers of patients in a short period of time. Now, with all health organizations cooperation, the situation is controlled. But the pandemic teaches the health system many lessons, and NOTA is one of these lessons.

Acute appendicitis is one of the most common surgical emergencies, and surgical management has been the gold standard for decades^3–7^. In adults and children, a high proportion of cases were treated successfully without surgery^3–9^. However, studies on children have lagged those on adults to some extent. In patients (over 5 years old) who presented with uncomplicated acute appendicitis, the success rate of medical treatment ranged between 88 and 90.8%^5,6^. Recently, there have been debates regarding the conservative antibiotic treatment for acute uncomplicated, and complicated appendicitis in children and adults. There are many studies suggesting the safety of antibiotic treatment alone^10–13^.

From this background, and since our surgical beds were converted to the COVID-19 care ward during the pandemic days, we adopted NOTA during the COVID-19 pandemic to decrease the pressure on the surgical ward and to face the shortage of beds for surgical patients. By adopting this project, NOTA decreased patient and hospital staff exposure to coronavirus during the pandemic situation, the possibility of viral pollution during laparoscopy was high, and it has been established that other viruses can be released during laparoscopy with carbon dioxide^1,2^. More added burden to the patients, staff, and resources was the PCR for coronavirus that was done obligatory for all patients presented with appendicitis before going to the OR as recommended by the health system. So, any positive COVID-19 case diagnosed as uncomplicated appendicitis was ideal to be involved in NOTA.

The aim of the study is to evaluate the cost-effectiveness and outcome of NOTA during the COVID-19 pandemic and to compare it to single-incision pediatric endo-surgery appendectomy (SIPESA). We used the opportunity of the COVID-19 pandemic to construct this prospective study to prove that NOTA is a feasible approach. The other aims were to reduce the length of hospital stays, decrease costs, and save hospital resources. We also want to reduce exposure risk and surgical complications, as well as the stress and psychological effects of surgery on parents and children.

## Methods

A prospective cohort study for NOTA of patients from 6 to 12 years old presented with a diagnosis of acute uncomplicated appendicitis in the COVID-19 pandemic period in our institute from April 1st, 2020, to April 30th, 2021. NOTA (non-operative treatment of acute appendicitis) was adopted if PAS was > 6, no fecolith or collection and symptoms less than 48 h. Surgeries were primarily performed for patients presented with acute appendicitis with fecolith or collection who presented late after 3–4 days or whose guardian refused the medical treatment.

Patients were divided into 2 groups: Group S was managed by surgery (SIPESA); Group N was managed by medical treatment (antibiotic).

We recommended the below clinical pathway for the management of our patients.

### Ethical approval and consent to participate

This study was approved by the Research Ethics Committee of King Fahad Armed Forces Hospital (KFAFH), Jeddah, Saudi Arabia. The parents or guardians of all participants provided written informed consent. The procedures used in this study adhere to the tenets of the Declaration of Helsinki.

### Clinical pathway: (Fig. 1)

**Figure 1:**
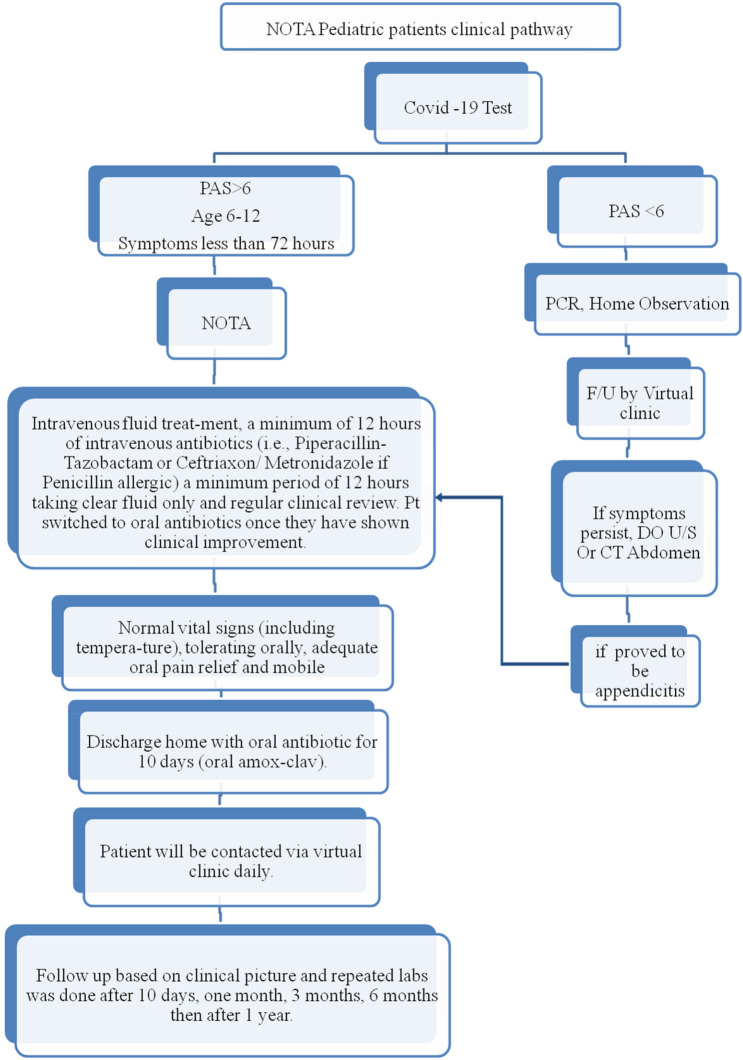
Clinical Pathway of the medical management of early acute appendicitis. NOTA: Non-operative treatment for simple acute appendicitis, Covid-19: Coronavirus, PAS: Pediatric Appendicitis Score, F/U: Follow up., U/S: Ultrasound, CT: Computerized Tomography.

1. The COVID-19 test was done routinely for the patients on admission to the emergency room during the pandemic, but now it is not routinely done.
2. If PAS (Pediatric Appendicitis Score) was < 6 (which will be explained below), the patient was advised to do home observations and follow up with a virtual clinic. But if symptoms persisted, an ultrasound or computerized tomography scan (if needed) was recommended.
3. NOTA (non-operative treatment of acute appendicitis) was adopted if PAS was > 6, age between 6 and 12 years, and symptoms less than 48 h. The treatment plan was intravenous fluid and a minimum of 12 h intravenous antibiotic (i.e., Piperacillin-Tazobactam) with a dose of ≤ 40 kg: 112.5 mg/kg (100 mg piperacillin/12.5 mg tazobactam) IV q8hr—> 40 kg: 3.375 g IV q6hr totaling 13.5 g (12 g piperacillin/1.5 g tazobactam)
4. After a minimum period of 12 h and a maximum of 72 h, the patient is allowed to take clear fluid only and undergo a regular clinical review. The patient was then switched to oral antibiotics once he or she had shown clinical improvement and was tolerating oral feeding.
5. The patient was discharged home once he had normal vital signs (including temperature) and was tolerating orally well. He was discharged home with oral antibiotics for 10 days (oral amoxiclav) at 40 mg/kg/day divided by 8 h.
6. The patient was contacted via virtual clinic daily. (This protocol was developed at the start of the study to support the patients and the staff and to give them confidence in the good results of the medical treatment.)
7. Follow-up was based on a clinical picture and repeated labs (only in the first visit) after 10 days, 1 month, 3 months, 6 months, and then 1 year.

The Pediatric Appendicitis Score (PAS)^14^ predicts the likelihood of appendicitis in pediatric patients (3–18 years old) with abdominal pain of ≤ 4 days duration. Stratifies patients into low-risk, high-risk, or equivocal for appendicitis. Includes findings from history, physical, and lab data. These variables were:

Symptoms (history):

1. Anorexia.
2. Pyrexia.
3. Nausea/emesis.
4. Migration of pain.

Signs (physical):

(5) Cough/percussion/hopping tenderness in the right lower quadrant of the abdomen.
(6) Tenderness over the right iliac fossa.

Investigations (labs):

(7) Leukocytosis.
(8) Polymorphonuclear neutrophilia.

Each of these variables was assigned a score of 1, except for physical signs (5 and 6), which were scored 2 to obtain a total of 10. We consider appendicitis if the score is more than 6, and we confirmed the diagnosis with ultrasound. If the score is less than 6 and symptoms persist, we also confirm the diagnosis by ultrasound or abdominal CT (if needed).

Visual analogue stress scale was used to measure the psychological effect on children. The scale ranged from 1 to 5, with number 1 doing great and number 5 cannot stand and ready to explode. The mean cost was calculated in the United States dollar as provided by the investment department on the basis that the package for laparoscopic appendectomy is $3900, while the cost of NOTA is $500 per day.

Data was collected by the pediatric surgeon who reviewed the closed chart who was responsible for discharging the patient home and the data was inserted in the Performa of the project designed in a Google sheet.

Descriptive statistics were carried out; categorical variables were summarized by number and percent, whereas continuous variables were summarized by the mean and standard deviation. The data displayed as a run chart to examine variation occurring at the aggregate level as well as linearity trend lines by using linear regression analysis to test a significant slope. All statistical analyses were performed using IBM Statistical Package for the Social Sciences statistics software, version 25 (SPSS, IBM Corp, Armonk, NY, USA). The pediatric surgery medical administration and research ethical committee approved this project. Consent from the patient was required for this project.

## Results

A total of 60 patients with uncomplicated simple acute appendicitis (early presentation in 48 h and no fecolith or collection) were managed from April 1st, 2020, to April 30th, 2021.

Group S (the surgical group) had 24 cases (40%), mean age 9.3 years. 16 patients (66%) were boys, and 8 (34%) were girls. Group N (the nonsurgical group) had 36 cases (60%), mean age 9.1 years. Twenty patients (55%) were boys and 16 (45%) were girls (Table 1).

**Table 1:** Summary of the comparison of demographic data between the study and control group.

Numbers of cases 60 (100%)		Surgical group (S) 24 (40%)	Nonsurgical group (N) 36(60%)	*p*-value
Mean age ± SD		9.3 ± 2.6 years	9.1 ± 3.1 years	0.486
Sex	Boys	16	20	0.385
	Girls	8	16	0.372

During the first 6 months of our study period, six cases (17%) were converted to surgical management. NOTA patients were secondarily converted to surgery after failure of medical treatment (persistence of symptoms for more than 72 h after NOTA started, worsening of symptoms, or development of complications). The intraoperative finding was that the appendicular inflammation was resolving. In the next 7 months, conversion was zero. We have four recurrences, which were defined as readmissions after discharge within 3 months with the same picture. The four cases operated upon admission.

Group N (nonsurgical group) had a significant decrease in the length of hospital stays (1.32 ± 0.72 days) compared to Group S (surgical group) (2.4 ± 1.35 days).

**Figure 2:**
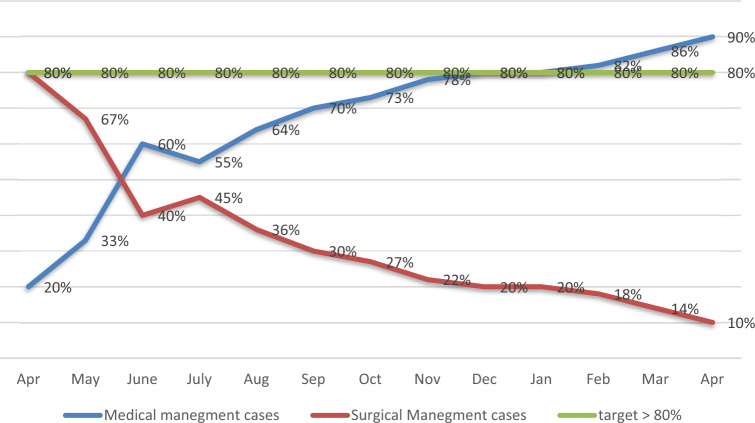
Percentage of Case allocation between surgical and medical management by month (with the target of 80% percent medical management).

**Figure 3:**
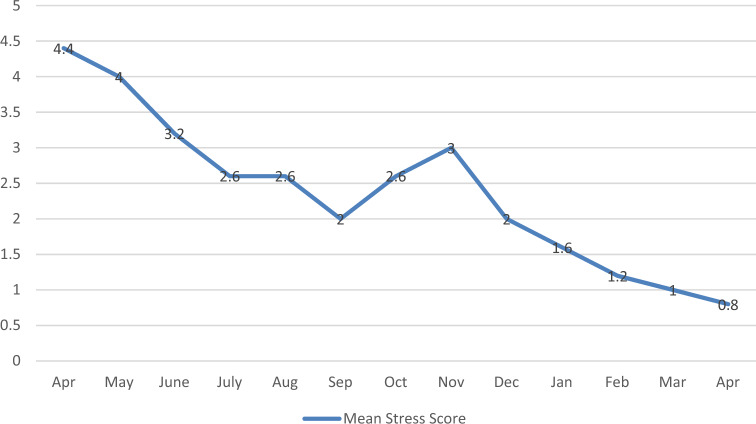
Psychological Stress for The Children (Visual analogue stress scale).

The case distribution per month in this period is shown in Fig. 2. Six cases (17%) in group N were converted to surgical management in the first 6 months of the study. The mean length of hospital stays dropped from 72 h in April 2020 to 18 h in April 2021. The mean psychological stress for the children improved from 4.4 to 0.8 (Fig. 3).

**Figure 4:**
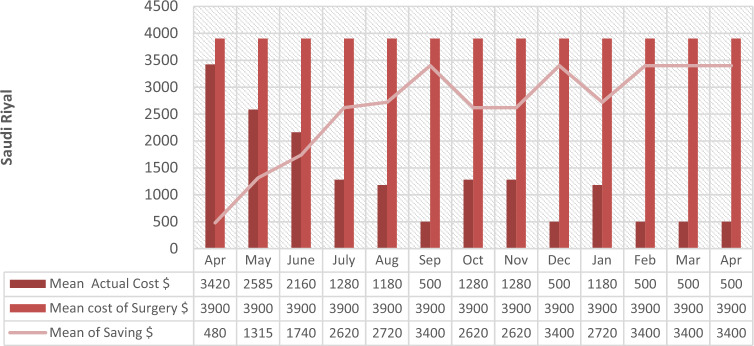
Mean Saving (surgical vs. medical management).

The follow-up of patients ranged from 1 to 6 months with a mean period of 3.5 months. The mean cost dropped from $2736/day to $400/day. The package for laparoscopic appendectomy is $3900, while the cost of NOTA is $500 per day. NOTA saves $3400 per case (Fig. 4).

## Discussion

The coronavirus disease 2019 (COVID-19) pandemic had a substantial effect on surgeons and patients who require surgical care. Providing care for patients with surgical disease requires a unique and intimate relationship between patients and surgeons, and this interaction and contact in many scenarios cannot be replaced by telehealth. As such, the surgical workforce has faced distinct challenges compared with nonsurgical specialties during the COVID-19 pandemic.

Our hospital implemented a new policy during the COVID-19 pandemic to protect the patient and the hospital staff and to limit the spread of the COVID-19 infection. This was done by providing personal protective equipment (PPE) such as masks and gowns, decreasing the length of hospital stay, saving intensive care unit (ICU) hospital beds and other resources needed for the care of hospitalized patients with the COVID-19 infection, and directing all available resources to control the COVID-19 pandemic.

Our hospital COVID-19 protective measures and staff reallocation in preponderance for surgery led to:

1. There is a shortage of hospital staff (anesthesia doctors, nursing staff, and surgical doctors) due to many reasons. As some of the staff were assigned to work on the COVID-19 team, the anesthesia doctors divided themselves into many teams to decrease exposure risk and isolate infected staff at home.
2. Shortage of hospital beds as the pediatric surgical beds were assigned to COVID-19 patients’ care and our surgical patients were admitted in the medical pediatric ward.
3. Preoperative preparation of urgent cases like acute appendicitis takes longer as COVID-19 PCR takes 9–72 h to be released at the start of the pandemic, but this duration has decreased to 1–2 h after that.
4. Lack of a CO_2_ filter in laparoscopic surgery changed our policy from laparoscopic to open procedure with its unsatisfactory outcome and drawbacks.

Therefore, we were obliged to apply the medical management for simple acute appendicitis after approval from the hospital ethical committee as the first-line treatment. The aim was to reduce the length of hospital stays, decrease costs, and save hospital resources. It also aimed to reduce exposure risk and surgical complications, decrease stress and the psychological effects of surgery on parents and children, and reduce the rate of negative appendectomies.

We faced many problems with the implementation of this modality of treatment: a small number of cases, a short period of follow-up, staff resistance, and a lack of experience and confidence. We overcame those problems by conducting many lectures with a review of recent literature, which strongly supported this modality of treatment with close observation and serial examination of patients during admission. Keeping patients in the hospital until we are sure that they are symptom-free and ready to go home with close follow-up at the virtual clinic. The length of hospital stay decreased gradually from 72 to 12 h due to the initial positive outcome of gaining confidence and experience in medical treatment.

Patients were discharged after a minimum of 12 h of intravenous antibiotics, and patients should tolerate oral feeding and antibiotics before being discharged home. During the first 6 months of our study period, there were six cases (17%) that converted to surgical management due to fear of complications and a lack of clinical judgement in medical management. The intraoperative finding was that the appendicular inflammation was resolving. In the next 7 months, conversion was zero. The follow-up of patients ranged from 1 to 6 months with a mean period of 3.5 months, and we had four recurrences and operated upon admission. These results showed that the application of the non-operative treatment increased the resolution of symptoms and the improvement of inflammatory markers. Besides, it decreases the psychological stress on children and families, especially in the situation of the COVID-19 pandemic. Certainly, this compares well with other reports applying non-operative management in acute appendicitis to be associated with a shorter hospital stay and a low risk of short-term recurrence^15–17^.

Though there is a report^15^ showing a 40% recurrence rate after 5 years of follow-up, likewise, Salminen et al., 2018 study^16^, which is an observational multicenter randomized clinical trial that also includes follow-up for 5 years, also showed a recurrence rate of 39.1% at 5 years. This obviously revealed the need for a second-phase follow-up to evaluate the role of non-operative therapy in treating acute appendicitis.

We strongly support NOTA to decrease costs, and it is a feasible modality of treatment for simple acute appendicitis in children. We decided to adopt NOTA in our center as the standard management of appendicitis in the pediatric age group, even after the COVID-19 pandemic. We found the results of our research promising, and NOTA significantly decreased hospital stays, costs, and psychological stress.

The study still has many limitations; it is a single-center study with a small number of cases and a short period of follow-up. Staff still have resistance, a lack of experience, and confidence in the new approach. Also, there is resistance from the guardian to NOTA, and they prefer the surgery over medical treatment. There is a lack of data on complications, readmission, recurrence, parents’ stress, etc. So, the conclusion on safety needs more follow-up and more cases.

## Conclusions

The lessons learned from the COVID-19 pandemic were that non-operative treatment of acute simple appendicitis in children is a feasible and cost-effective approach with less psychological stress on patients and parents.

## Data Availability

The datasets used and/or analysed during the current study are available from the corresponding author on reasonable request.

## Abbreviations

NOTA: Non-operative treatment for simple acute appendicitis
COVID-19: Coronavirus
SIPESA: Single incision pediatric endo-surgery appendectomy
PCR: Polymerase chain reaction
PAS: Pediatric appendicitis score
